# Crystal structure of [{FeCl_3_}_2_(μ-PC^H^P)_2_] [PC^H^P = 1,3-bis­(2-di­phenyl­phosphanyleth­yl)-3*H*-imidazol-1-ium] with an unknown solvent

**DOI:** 10.1107/S205698901801472X

**Published:** 2018-10-31

**Authors:** Nadja Stucke, Christian Näther, Felix Tuczek

**Affiliations:** a Institut für Anorganische Chemie, Universität Kiel, Max-Eyth-Str. 2, 24118, Kiel, Germany

**Keywords:** crystal structure, iron(II) tri­chlorido complex, phosphine ligands

## Abstract

The crystal structure of the title compound, bis­{μ-1,3-bis­[2-(di­phenyl­phosphan­yl)eth­yl]-1*H*-imidazole-κ^2^ *P*:*P*′}bis­[tri­chlorido­iron(III)], [Fe_2_Cl_6_(C_31_H_31_N_2_P_2_)_2_] or [{FeCl_3_}_2_(μ-PC^H^P)_2_] (PC^H^P = C_31_H_31_N_2_P_2_), consists of dinuclear complexes that are located about centres of inversion. The Fe^III^ cation is in a distorted trigonal–bipyramidal coordination with three chloride ligands located in the trigonal plane and two P atoms of symmetry-related PC^H^P ligands occupying the axial positions. Within the centrosymmetric complex, a pair of intra­molecular C—H⋯Cl hydrogen bonds between aromatic CH groups and chloride ligands are found. Individual complexes are linked into layers parallel to (01) by inter­molecular C—H⋯Cl hydrogen bonds. No pronounced inter­molecular inter­actions occur between these layers. This arrangement leaves space for disordered solvent mol­ecules. Electron density associated with these additional solvent mol­ecules was removed with the SQUEEZE procedure in *PLATON* [Spek (2015 ▸). *Acta Cryst.* C**71**, 9–18]. The given chemical formula and other crystal data do not take into account the unknown solvent mol­ecule(s).

## Abstract 2

In the crystal structure of the title compound, binuclear centrosymmetric mol­ecules are present. The Fe^III^ cation exhibits a trigonal–bipyramidal environment, being coordinated by three Cl ligands in the equatorial plane and two P atoms of symmetry-related PC^H^P ligands at the axial sites. The complex mol­ecules are linked into layers by inter­molecular C—H⋯Cl hydrogen bonding.

## Chemical context   

The conversion of di­nitro­gen into ammonia is an inter­esting reaction in the area of bioinorganic chemistry. In nature, the enzyme nitro­genase comprising the iron molybdenum cofactor (an MoFe_7_S_9_C-cluster), catalyses the derivatization of di­nitro­gen (Burgess & Lowe, 1996 ▸; Spatzal *et al.*, 2011 ▸; Lancaster *et al.*, 2011 ▸). Based on spectroscopic, biochemical and theoretical investigations, one of the iron atoms of the MoFe cofactor is considered to be the binding site of the di­nitro­gen mol­ecule (Hoffman *et al.*, 2009 ▸, 2014 ▸). For this reason, the synthesis of model systems based on iron complexes serving as N_2_ → NH_3_ catalysts has gained in importance over the past few years (Stucke *et al.*, 2018 ▸). In particular, iron(II) di­nitro­gen complexes containing a PCP pincer ligand with a central *N*-heterocyclic carbene (Lee *et al.*, 2004 ▸) are of significant inter­est because they are able to bind and activate di­nitro­gen. As a result of the strong *σ*-donor property of the central carbene unit, electron density is transferred to the central metal atom and to the N_2_ ligand (Gradert *et al.*, 2015 ▸). In this way, the di­nitro­gen mol­ecule coordinating to the iron(II) cation should be activated sufficiently in order to get protonated, which is the first step in the N_2_ → NH_3_ conversion (Yandulov & Schrock, 2003 ▸; Del Castillo *et al.*, 2016 ▸)

In this context we are inter­ested in the synthesis of iron di­nitro­gen complexes containing PCP pincer ligands. In the course of this project we serendipitously obtained crystals of the title compound by the reaction of the PC^H^P pincer ligand and the dinuclear iron(II) precursor [{FeCl(tmeda)}_2_(μ-Cl)_2_]. To prove the identity of this compound, a single crystal X-ray structure determination was performed, which revealed that the central carbene C atom is protonated and that a dimeric iron(II) tri­chlorido complex has formed. Comparison of the experimental X-ray powder diffraction pattern with the calculated pattern on the basis of single-crystal data shows that the obtained product contained the title compound as the major phase but is contaminated with small amounts of other unknown crystalline phase(s) (Supplementary Fig. S1).
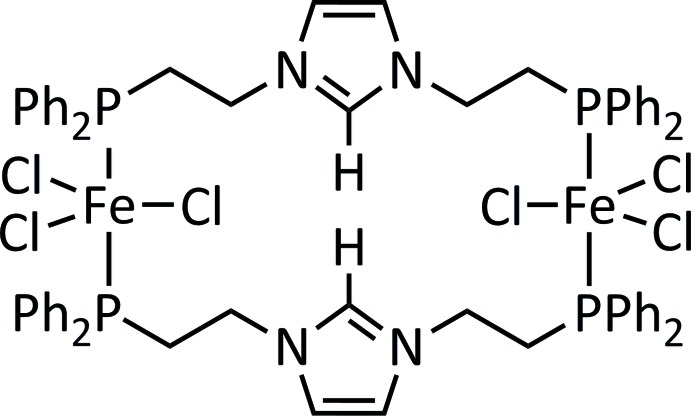


## Structural commentary   

**Figure 1:**
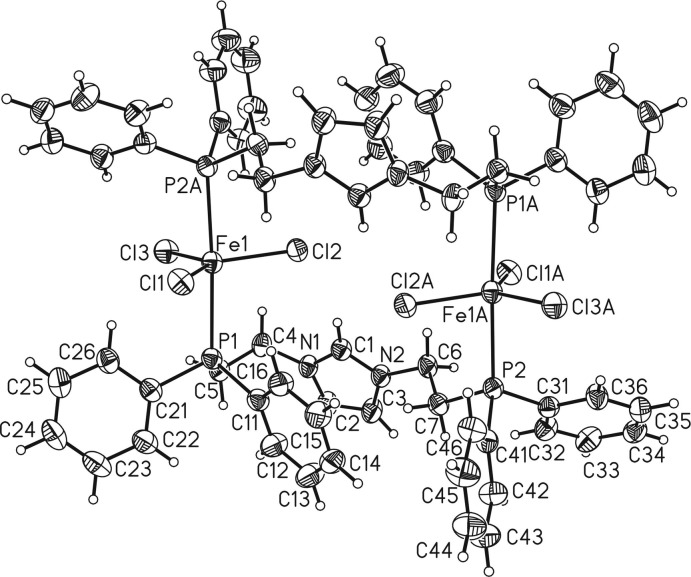
Mol­ecular structure of the title compound with atom labelling and displacement ellipsoids drawn at the 50% probability level. [Symmetry code: (*A*) –*x* + 1, –*y* + 1, –*z* + 1.]

The asymmetric unit of the title compound consists of one Fe^III^ cation, three chlorido ligands and one PC^H^P ligand. The binuclear mol­ecule is completed by inversion symmetry. The Fe^III^ cation has a distorted trigonal–bipyramidal environment, being coordinated by two phospho­rus atoms of two symmetry-related PC^H^P ligands that occupy the axial positions and by three chlorido ligands that are located in the trigonal plane of the bipyramid (Figs. 1 ▸ and 2 ▸). The Fe—Cl bond lengths range from 2.3193 (5) to 2.3499 (4) Å and are much shorter than the Fe—P bond lengths of 2.6014 (5) and 2.6329 (5) Å (Table 1 ▸). In the binuclear mol­ecule, the two iron(II) cations are linked by pairs of PC^H^P ligands (Figs. 1 ▸, 2 ▸). The protonation of the central carbene moiety and hence the +2 oxidation state of iron of was proven by localization of the H atom attached to C1 and free refinement of its position. We also looked for tri­chlorido iron complexes with a trigonal–bipyramidal configuration in which the central iron atom has an oxidation state of +3. In comparison with the title compound, the Fe—Cl bond lengths in these complexes are significantly shorter (2.21 to 2.27 Å; Walker & Poli, 1989 ▸; Feng *et al.*, 2017 ▸), thus confirming the oxidation state +2 of the iron cation in [{FeCl_3_}_2_(μ-PC^H^P)_2_].

**Figure 2:**
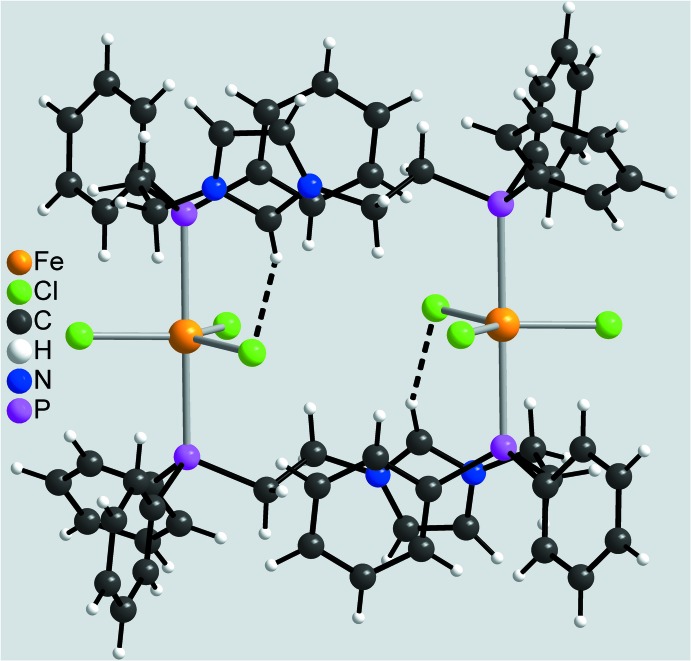
Mol­ecular structure of the title compound showing the intra­molecular C—H⋯Cl hydrogen bonds as as dashed lines. For clarity, only the hydrogen bonds with short H⋯Cl distances (C1—H1⋯Cl2) are shown.

Finally it is noted that within the dimer, a pair of intra­molecular C—H⋯Cl hydrogen bonds between the aromatic H atom attached to C1 and one of the chlorido ligands is observed (Fig. 2 ▸, Table 2 ▸). There is an additional intra­molecular contact between the H atom attached to C16 and Cl1, but at a much longer H⋯Cl distance (Table 2 ▸).

## Supra­molecular features   

**Figure 3:**
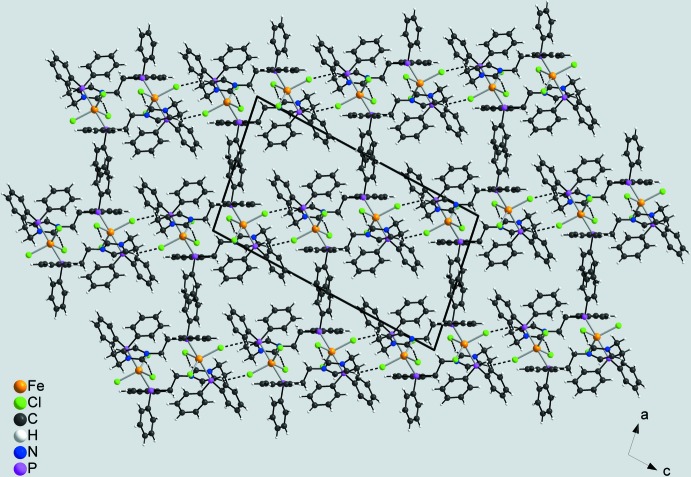
Crystal structure of the title compound in a view along [010]. Inter­molecular C—H⋯Cl hydrogen bonds are shown as dashed lines. For clarity, only the short hydrogen bond with a H⋯Cl distance of 2.65 Å is shown.

**Figure 4:**
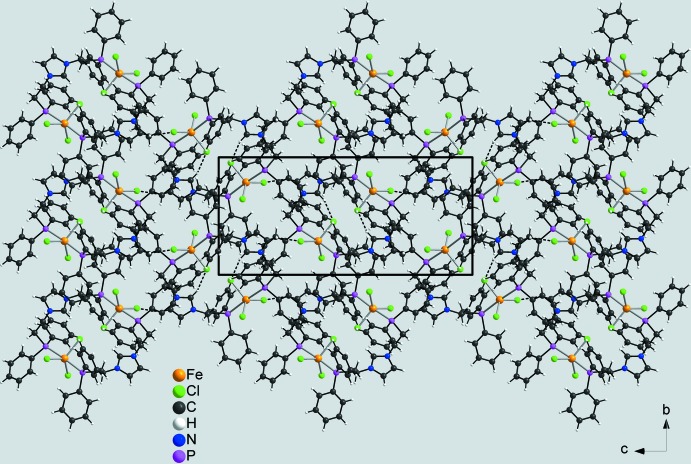
Crystal structure of the title compound in a view along [100]. For clarity, only short intra- and inter­molecular C—H⋯Cl hydrogen bonds with H⋯Cl distances of 2.43 and 2.65 Å, respectively, are shown as dashed lines.

In the crystal structure, the dimers are linked by centrosymmetric pairs of C—H⋯Cl hydrogen bonds between the H atom attached to C2 and the Cl3 atom of a neighbouring complex into layers parallel to (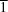
01) (Fig. 3 ▸, Table 2 ▸). Within these layers there are a number of additional C—H⋯Cl contacts, but either at much longer H⋯Cl distances or with angles deviating strongly from linearity (Table 2 ▸). These layers are stacked along [100] with no pronounced inter­molecular inter­actions between them (Fig. 4 ▸, Table 2 ▸). By this arrangement, large cavities are formed in which disordered solvent mol­ecules of unknown identity are present (see *Refinement*).

## Database survey   

To the best of our knowledge, no other iron complexes with the PC^H^P ligand have been reported in the literature. However, a few iron complexes where iron is coordinated by three chlorido and two phosphine ligands in a trigonal–bipyramidal environment are known (Walker & Poli, 1989 ▸; Feng *et al.*, 2017 ▸). Furthermore, other metal complexes of silver, palladium, rhodium and molybdenum with the metal coordinated by the deprotonated PC^H^P ligand have been reported and are well investigated (Lee *et al.*, 2004 ▸; Zeng *et al.*, 2005 ▸; Gradert *et al.*, 2013 ▸). The difference between these complexes and the title complex [{FeCl_3_}_2_(μ-PC^H^P)_2_] is the coordination of the carbene unit to the central metal cation, leading to the formation of mononuclear complexes. Nevertheless, a dinuclear gold complex with two bridging PC^H^P ligands was obtained by Bestgen *et al.* (2015 ▸). Here, the PC^H^P pincer ligands exhibit the same coordination mode as in the title complex [{FeCl_3_}_2_(μ-PC^H^P)_2_], *i.e.* the pincer ligand binds to the central metal merely with the two phosphine donor groups. Polynuclear silver complexes with the PC^H^P ligand have also been reported, but in contrast to the aforementioned gold complex the central carbene unit does coordinate to the silver atom (Chiu *et al.*, 2005 ▸).

## Synthesis and crystallization   

Synthetic procedures were performed according to Xiang *et al.* (2011 ▸). To 230 mg (435 µmol) of 1,3-bis­(2-di­phenyl­phos­phan­yl­eth­yl)-3*H*-imidazol-1-ium chloride (PC^H^P·Cl), which was prepared according to literature procedures (Lee *et al.*, 2004 ▸), and 54.0 mg (482 µmol) of KO^*t*^Bu was added toluene (20 ml). The mixture was stirred at room temperature for 2 h. Afterwards, the suspension was filtered and added to 100 mg (207 µmol) of [{FeCl(tmeda)}_2_(μ-Cl)_2_] in 5 ml of toluene. The iron complex had been prepared according to a literature protocol (Davies *et al.*, 1997 ▸). After the reaction mixture had been stirred at room temperature overnight, the solution was concentrated under vacuum to 15 ml. The precipitate was filtered off, washed with toluene and dried under vacuum. The product was obtained as a light-brown solid (145 mg). Colourless crystals suitable for single-crystal X-ray diffraction were grown by diffusion of diethyl ether into a methanol solution of the product. Presumably, the protonation of the central carbene unit results from the crystallization process in protic methanol.

## Refinement   

Crystal data, data collection and structure refinement details are summarized in Table 3 ▸. The C—H hydrogen atoms were located in difference maps but were refined using a riding model with idealized positions [*U*_iso_(H) = 1.2*U*_eq_(C) with C—H = 0.95 Å for aromatic and 0.99 Å for methyl­ene H atoms]. In the first stage of structure refinement, the hydrogen atom bound to the carbene C1 atom was clearly discernible in a difference map and was refined with varying coordinates and varying isotropic displacement parameters to prove that the carbene C atom is definitely protonated. Some very weak residual electron density peaks were present after the final refinement, indicating disordered solvent mol­ecules. Since the disorder could not be resolved by various split models and the nature and number of solvent mol­ecules (diethyl ether, methanol) could not be determined, all electron density associated with the solvent mol­ecule(s) was removed using the SQUEEZE procedure in *PLATON* (Spek, 2015 ▸). The volume of the solvent-accessible voids amounts to 734.2 Å^3^ per unit cell; the calculated number of electrons within the voids is 173.4. The given chemical formula and other crystal data do not take into account the unknown solvent mol­ecule(s).

## Supplementary Material

Crystal structure: contains datablock(s) I. DOI: 10.1107/S205698901801472X/wm5467sup1.cif

Structure factors: contains datablock(s) I. DOI: 10.1107/S205698901801472X/wm5467Isup2.hkl

Experimental (top) and calculated (bottom) X-ray powder pattern for the title compound. Note that the shift of the reflections to lower Bragg angles for the experimental pattern can be traced back to the fact that the powder pattern was measured at room-temperature while the simulated pattern uses the low temperature data of the current single crystal determination.. DOI: 10.1107/S205698901801472X/wm5467sup4.jpg

CCDC reference: 1874120

Additional supporting information:  crystallographic information; 3D view; checkCIF report

## Figures and Tables

**Table 1:** Selected geometric parameters (Å, °)

Fe1—Cl1	2.3193 (5)	Fe1—P2^i^	2.6014 (5)
Fe1—Cl2	2.3285 (5)	Fe1—P1	2.6329 (5)
Fe1—Cl3	2.3499 (4)		
			
Cl1—Fe1—Cl2	119.70 (2)	Cl3—Fe1—P2^i^	92.918 (16)
Cl1—Fe1—Cl3	127.439 (19)	Cl1—Fe1—P1	87.544 (16)
Cl2—Fe1—Cl3	112.83 (2)	Cl2—Fe1—P1	96.663 (17)
Cl1—Fe1—P2^i^	88.188 (16)	Cl3—Fe1—P1	88.327 (16)
Cl2—Fe1—P2^i^	86.935 (16)	P2^i^—Fe1—P1	175.405 (17)

Symmetry code: (i) 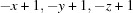.

**Table 2:** Hydrogen-bond geometry (Å, °)

*D*—H⋯*A*	*D*—H	H⋯*A*	*D*⋯*A*	*D*—H⋯*A*
C2—H2⋯Cl3^ii^	0.95	2.65	3.4710 (18)	145
C3—H3⋯Cl1^iii^	0.95	2.80	3.406 (2)	123
C4—H4*A*⋯Cl3^ii^	0.99	2.98	3.6985 (18)	130
C6—H6*A*⋯Cl1^i^	0.99	2.77	3.4402 (17)	126
C6—H6*B*⋯Cl1^iii^	0.99	2.84	3.6716 (19)	143
C16—H16⋯Cl1	0.95	2.93	3.726 (2)	143
C32—H32⋯Cl1^iii^	0.95	2.91	3.6057 (17)	131
C1—H1⋯Cl2	0.95	2.43	3.3260 (17)	157

Symmetry codes: (i) 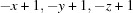; (ii) 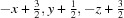; (iii) 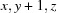.

**Table 3:** Experimental details

Crystal data	
Chemical formula	[Fe_2_Cl_6_(C_31_H_31_N_2_P_2_)_2_]
*M*_r_	1311.44
Crystal system, space group	Monoclinic, *P*2_1_/*n*
Temperature (K)	170
*a*, *b*, *c* (Å)	13.5685 (3), 11.0227 (1), 24.1575 (5)
β (°)	100.142 (2)
*V* (Å^3^)	3556.58 (11)
*Z*	2
Radiation type	Mo *K*α
μ (mm^−1^)	0.76
Crystal size (mm)	0.15 × 0.12 × 0.07

Data collection	
Diffractometer	Stoe IPDS2
Absorption correction	Numerical (*X-RED* and *X-SHAPE*; Stoe & Cie, 2008 ▸)
*T*_min_, *T*_max_	0.840, 0.949
No. of measured, independent and observed [*I* > 2σ(*I*)] reflections	54486, 8468, 7787
*R*_int_	0.037
(sin θ/λ)_max_ (Å^−1^)	0.659

Refinement	
*R*[*F* ^2^ > 2σ(*F* ^2^)], *wR*(*F* ^2^), *S*	0.036, 0.087, 1.07
No. of reflections	8468
No. of parameters	352
H-atom treatment	H-atom parameters constrained
Δρ_max_, Δρ_min_ (e Å^−3^)	0.37, −0.34

Computer programs: *X-AREA* (Stoe & Cie, 2008 ▸), *SHELXS97* (Sheldrick, 2008 ▸), *SHELXL2014* (Sheldrick, 2015 ▸), *XP* (Sheldrick, 2008 ▸), *DIAMOND* (Brandenburg, 2014 ▸) and *publCIF* (Westrip, 2010 ▸).

## supplementary crystallographic information

### Crystal data

**Table:**

[Fe_2_Cl_6_(C_31_H_31_N_2_P_2_)_2_]	*F*(000) = 1352
*M**_r_* = 1311.44	*D*_x_ = 1.225 Mg m^−^^3^
Monoclinic, *P*2_1_/*n*	Mo *K*α radiation, λ = 0.71073 Å
*a* = 13.5685 (3) Å	Cell parameters from 54486 reflections
*b* = 11.0227 (1) Å	θ = 1.7–28.0°
*c* = 24.1575 (5) Å	µ = 0.76 mm^−^^1^
β = 100.142 (2)°	*T* = 170 K
*V* = 3556.58 (11) Å^3^	Block, colorless
*Z* = 2	0.15 × 0.12 × 0.07 mm

### Data collection

**Table:**

Stoe IPDS-2 diffractometer	7787 reflections with *I* > 2σ(*I*)
ω scans	*R*_int_ = 0.037
Absorption correction: numerical (X-RED and X-SHAPE; Stoe & Cie, 2008)	θ_max_ = 28.0°, θ_min_ = 1.7°
*T*_min_ = 0.840, *T*_max_ = 0.949	*h* = −17→17
54486 measured reflections	*k* = −13→14
8468 independent reflections	*l* = −31→31

### Refinement

**Table:**

Refinement on *F*^2^	0 restraints
Least-squares matrix: full	Hydrogen site location: inferred from neighbouring sites
*R*[*F*^2^ > 2σ(*F*^2^)] = 0.036	H-atom parameters constrained
*wR*(*F*^2^) = 0.087	*w* = 1/[σ^2^(*F*_o_^2^) + (0.0386*P*)^2^ + 1.7166*P*] where *P* = (*F*_o_^2^ + 2*F*_c_^2^)/3
*S* = 1.07	(Δ/σ)_max_ = 0.002
8468 reflections	Δρ_max_ = 0.37 e Å^−^^3^
352 parameters	Δρ_min_ = −0.34 e Å^−^^3^

### Special details

**Table:**

Geometry. All esds (except the esd in the dihedral angle between two l.s. planes) are estimated using the full covariance matrix. The cell esds are taken into account individually in the estimation of esds in distances, angles and torsion angles; correlations between esds in cell parameters are only used when they are defined by crystal symmetry. An approximate (isotropic) treatment of cell esds is used for estimating esds involving l.s. planes.

### Fractional atomic coordinates and isotropic or equivalent isotropic displacement parameters (Å^2^)

**Table:**

	*x*	*y*	*z*	*U*_iso_*/*U*_eq_	
Fe1	0.63910 (2)	0.28699 (2)	0.60806 (2)	0.03183 (7)	
Cl1	0.51169 (3)	0.14406 (4)	0.59404 (2)	0.04101 (10)	
Cl2	0.63279 (4)	0.45216 (4)	0.54744 (2)	0.04799 (12)	
Cl3	0.78456 (3)	0.28478 (4)	0.67715 (2)	0.04270 (11)	
C1	0.55808 (12)	0.69486 (15)	0.61066 (7)	0.0340 (3)	
H1	0.5664	0.6344	0.5839	0.041*	
N1	0.59768 (10)	0.69243 (13)	0.66511 (6)	0.0343 (3)	
C2	0.56772 (15)	0.79492 (17)	0.69012 (7)	0.0445 (4)	
H2	0.5846	0.8159	0.7288	0.053*	
C3	0.50986 (15)	0.86009 (17)	0.64948 (7)	0.0448 (4)	
H3	0.4785	0.9356	0.6541	0.054*	
N2	0.50507 (11)	0.79597 (13)	0.60010 (6)	0.0350 (3)	
C4	0.66873 (12)	0.60071 (16)	0.69286 (7)	0.0367 (3)	
H4A	0.7229	0.6424	0.7187	0.044*	
H4B	0.6995	0.5588	0.6639	0.044*	
C5	0.62115 (12)	0.50624 (16)	0.72590 (7)	0.0351 (3)	
H5A	0.5782	0.5484	0.7489	0.042*	
H5B	0.6749	0.4643	0.7520	0.042*	
C6	0.45503 (12)	0.83335 (16)	0.54351 (6)	0.0360 (3)	
H6A	0.4809	0.7843	0.5149	0.043*	
H6B	0.4704	0.9196	0.5373	0.043*	
C7	0.34257 (12)	0.81690 (16)	0.53670 (7)	0.0355 (3)	
H7A	0.3158	0.8778	0.5603	0.043*	
H7B	0.3286	0.7356	0.5509	0.043*	
P1	0.54491 (3)	0.39123 (4)	0.68183 (2)	0.03175 (9)	
C11	0.42554 (12)	0.46751 (16)	0.65615 (7)	0.0355 (3)	
C12	0.38523 (15)	0.5591 (2)	0.68493 (8)	0.0479 (4)	
H12	0.4205	0.5857	0.7203	0.057*	
C13	0.29422 (16)	0.6119 (2)	0.66253 (9)	0.0551 (5)	
H13	0.2674	0.6740	0.6827	0.066*	
C14	0.24243 (14)	0.5746 (2)	0.61094 (8)	0.0470 (4)	
H14	0.1793	0.6094	0.5961	0.056*	
C15	0.28280 (14)	0.4868 (2)	0.58128 (8)	0.0466 (4)	
H15	0.2481	0.4625	0.5454	0.056*	
C16	0.37388 (13)	0.43355 (18)	0.60342 (7)	0.0418 (4)	
H16	0.4012	0.3733	0.5824	0.050*	
C21	0.51823 (12)	0.28897 (16)	0.73749 (7)	0.0364 (3)	
C22	0.45819 (15)	0.3229 (2)	0.77591 (8)	0.0468 (4)	
H22	0.4249	0.3991	0.7720	0.056*	
C23	0.44646 (16)	0.2461 (2)	0.82003 (9)	0.0548 (5)	
H23	0.4057	0.2702	0.8463	0.066*	
C24	0.49388 (16)	0.1350 (2)	0.82568 (9)	0.0548 (5)	
H24	0.4860	0.0828	0.8559	0.066*	
C25	0.55305 (16)	0.0996 (2)	0.78729 (9)	0.0510 (5)	
H25	0.5854	0.0228	0.7910	0.061*	
C26	0.56500 (14)	0.17639 (18)	0.74351 (8)	0.0412 (4)	
H26	0.6057	0.1518	0.7173	0.049*	
P2	0.27552 (3)	0.83192 (4)	0.46365 (2)	0.03108 (9)	
C31	0.25966 (12)	0.99424 (15)	0.45051 (7)	0.0329 (3)	
C32	0.29258 (13)	1.08429 (17)	0.48956 (8)	0.0410 (4)	
H32	0.3247	1.0628	0.5264	0.049*	
C33	0.27869 (14)	1.20583 (18)	0.47496 (9)	0.0467 (4)	
H33	0.3025	1.2668	0.5018	0.056*	
C34	0.23076 (14)	1.23876 (17)	0.42205 (9)	0.0435 (4)	
H34	0.2199	1.3220	0.4127	0.052*	
C35	0.19850 (15)	1.14984 (18)	0.38268 (8)	0.0455 (4)	
H35	0.1658	1.1720	0.3460	0.055*	
C36	0.21367 (15)	1.02863 (17)	0.39651 (7)	0.0421 (4)	
H36	0.1926	0.9682	0.3689	0.051*	
C41	0.15144 (13)	0.78454 (16)	0.47522 (7)	0.0360 (3)	
C42	0.09715 (15)	0.85216 (19)	0.50838 (9)	0.0469 (4)	
H42	0.1225	0.9278	0.5235	0.056*	
C43	0.00641 (16)	0.8097 (2)	0.51940 (10)	0.0557 (5)	
H43	−0.0302	0.8560	0.5420	0.067*	
C44	−0.03052 (16)	0.7005 (2)	0.49754 (10)	0.0584 (6)	
H44	−0.0927	0.6714	0.5051	0.070*	
C45	0.02225 (17)	0.6330 (2)	0.46464 (10)	0.0568 (5)	
H45	−0.0038	0.5575	0.4497	0.068*	
C46	0.11314 (14)	0.67456 (18)	0.45325 (8)	0.0443 (4)	
H46	0.1491	0.6278	0.4304	0.053*	

### Atomic displacement parameters (Å^2^)

**Table:**

	*U*^11^	*U*^22^	*U*^33^	*U*^12^	*U*^13^	*U*^23^
Fe1	0.03217 (12)	0.03458 (13)	0.02699 (11)	−0.00087 (9)	0.00044 (8)	−0.00086 (9)
Cl1	0.0426 (2)	0.0440 (2)	0.0352 (2)	−0.00998 (17)	0.00339 (16)	−0.00436 (16)
Cl2	0.0718 (3)	0.0384 (2)	0.0373 (2)	0.0130 (2)	0.0192 (2)	0.00647 (17)
Cl3	0.0369 (2)	0.0575 (3)	0.02998 (19)	0.00243 (18)	−0.00435 (15)	−0.00324 (17)
C1	0.0352 (8)	0.0361 (8)	0.0286 (7)	−0.0002 (6)	0.0004 (6)	−0.0016 (6)
N1	0.0364 (7)	0.0341 (7)	0.0294 (6)	0.0017 (6)	−0.0029 (5)	0.0011 (5)
C2	0.0590 (11)	0.0406 (9)	0.0289 (8)	0.0068 (8)	−0.0060 (7)	−0.0050 (7)
C3	0.0604 (11)	0.0392 (9)	0.0310 (8)	0.0110 (8)	−0.0026 (8)	−0.0040 (7)
N2	0.0390 (7)	0.0374 (7)	0.0258 (6)	0.0033 (6)	−0.0023 (5)	−0.0004 (5)
C4	0.0326 (8)	0.0376 (8)	0.0364 (8)	0.0004 (7)	−0.0038 (6)	0.0037 (7)
C5	0.0362 (8)	0.0393 (8)	0.0272 (7)	0.0025 (7)	−0.0017 (6)	−0.0003 (6)
C6	0.0385 (8)	0.0417 (9)	0.0244 (7)	0.0027 (7)	−0.0036 (6)	0.0020 (6)
C7	0.0386 (8)	0.0408 (9)	0.0255 (7)	0.0022 (7)	0.0013 (6)	−0.0002 (6)
P1	0.03150 (19)	0.0367 (2)	0.02601 (18)	0.00026 (16)	0.00210 (14)	0.00051 (15)
C11	0.0336 (8)	0.0417 (9)	0.0308 (8)	0.0006 (7)	0.0045 (6)	0.0016 (7)
C12	0.0437 (10)	0.0584 (12)	0.0391 (9)	0.0106 (9)	0.0006 (7)	−0.0086 (8)
C13	0.0479 (11)	0.0642 (13)	0.0519 (11)	0.0175 (10)	0.0055 (9)	−0.0080 (10)
C14	0.0342 (8)	0.0597 (12)	0.0461 (10)	0.0093 (8)	0.0048 (7)	0.0047 (9)
C15	0.0383 (9)	0.0598 (12)	0.0384 (9)	0.0017 (8)	−0.0025 (7)	−0.0002 (8)
C16	0.0385 (9)	0.0491 (10)	0.0360 (8)	0.0041 (8)	0.0017 (7)	−0.0039 (7)
C21	0.0344 (8)	0.0431 (9)	0.0302 (8)	−0.0043 (7)	0.0014 (6)	0.0013 (7)
C22	0.0449 (10)	0.0556 (11)	0.0418 (10)	−0.0023 (8)	0.0123 (8)	0.0028 (8)
C23	0.0510 (11)	0.0728 (14)	0.0441 (10)	−0.0108 (10)	0.0175 (9)	0.0049 (10)
C24	0.0535 (11)	0.0652 (13)	0.0452 (10)	−0.0149 (10)	0.0073 (9)	0.0167 (10)
C25	0.0522 (11)	0.0494 (11)	0.0488 (11)	−0.0055 (9)	0.0020 (9)	0.0129 (9)
C26	0.0408 (9)	0.0458 (10)	0.0355 (8)	−0.0025 (8)	0.0024 (7)	0.0052 (7)
P2	0.03107 (19)	0.0346 (2)	0.02607 (18)	0.00111 (15)	0.00082 (14)	−0.00094 (15)
C31	0.0301 (7)	0.0356 (8)	0.0321 (7)	0.0008 (6)	0.0033 (6)	−0.0015 (6)
C32	0.0381 (8)	0.0426 (9)	0.0384 (9)	0.0021 (7)	−0.0037 (7)	−0.0054 (7)
C33	0.0417 (9)	0.0389 (9)	0.0565 (11)	0.0006 (7)	0.0007 (8)	−0.0098 (8)
C34	0.0407 (9)	0.0352 (9)	0.0560 (11)	0.0023 (7)	0.0125 (8)	0.0038 (8)
C35	0.0512 (10)	0.0451 (10)	0.0395 (9)	0.0064 (8)	0.0064 (8)	0.0071 (8)
C36	0.0529 (10)	0.0395 (9)	0.0319 (8)	0.0021 (8)	0.0020 (7)	−0.0007 (7)
C41	0.0350 (8)	0.0396 (9)	0.0325 (8)	0.0017 (7)	0.0034 (6)	0.0024 (7)
C42	0.0443 (10)	0.0476 (11)	0.0513 (11)	0.0003 (8)	0.0157 (8)	−0.0051 (8)
C43	0.0471 (11)	0.0623 (13)	0.0622 (13)	0.0046 (10)	0.0218 (10)	−0.0033 (10)
C44	0.0430 (10)	0.0674 (14)	0.0682 (14)	−0.0082 (10)	0.0190 (10)	0.0003 (11)
C45	0.0531 (11)	0.0568 (12)	0.0632 (13)	−0.0166 (10)	0.0174 (10)	−0.0089 (10)
C46	0.0432 (9)	0.0472 (10)	0.0435 (9)	−0.0059 (8)	0.0106 (8)	−0.0060 (8)

### Geometric parameters (Å, º)

**Table:**

Fe1—Cl1	2.3193 (5)	C15—H15	0.9500
Fe1—Cl2	2.3285 (5)	C16—H16	0.9500
Fe1—Cl3	2.3499 (4)	C21—C22	1.390 (3)
Fe1—P2^i^	2.6014 (5)	C21—C26	1.390 (3)
Fe1—P1	2.6329 (5)	C22—C23	1.392 (3)
C1—N2	1.327 (2)	C22—H22	0.9500
C1—N1	1.331 (2)	C23—C24	1.379 (3)
C1—H1	0.9500	C23—H23	0.9500
N1—C2	1.376 (2)	C24—C25	1.385 (3)
N1—C4	1.474 (2)	C24—H24	0.9500
C2—C3	1.351 (2)	C25—C26	1.387 (3)
C2—H2	0.9500	C25—H25	0.9500
C3—N2	1.378 (2)	C26—H26	0.9500
C3—H3	0.9500	P2—C31	1.8233 (17)
N2—C6	1.4740 (19)	P2—C41	1.8306 (18)
C4—C5	1.523 (2)	P2—Fe1^i^	2.6013 (5)
C4—H4A	0.9900	C31—C32	1.388 (2)
C4—H4B	0.9900	C31—C36	1.396 (2)
C5—P1	1.8500 (17)	C32—C33	1.390 (3)
C5—H5A	0.9900	C32—H32	0.9500
C5—H5B	0.9900	C33—C34	1.377 (3)
C6—C7	1.517 (2)	C33—H33	0.9500
C6—H6A	0.9900	C34—C35	1.382 (3)
C6—H6B	0.9900	C34—H34	0.9500
C7—P2	1.8450 (16)	C35—C36	1.384 (3)
C7—H7A	0.9900	C35—H35	0.9500
C7—H7B	0.9900	C36—H36	0.9500
P1—C11	1.8334 (17)	C41—C46	1.387 (3)
P1—C21	1.8387 (18)	C41—C42	1.395 (3)
C11—C12	1.391 (3)	C42—C43	1.386 (3)
C11—C16	1.393 (2)	C42—H42	0.9500
C12—C13	1.386 (3)	C43—C44	1.373 (3)
C12—H12	0.9500	C43—H43	0.9500
C13—C14	1.381 (3)	C44—C45	1.378 (3)
C13—H13	0.9500	C44—H44	0.9500
C14—C15	1.373 (3)	C45—C46	1.388 (3)
C14—H14	0.9500	C45—H45	0.9500
C15—C16	1.388 (3)	C46—H46	0.9500
			
Cl1—Fe1—Cl2	119.70 (2)	C14—C15—C16	120.46 (18)
Cl1—Fe1—Cl3	127.439 (19)	C14—C15—H15	119.8
Cl2—Fe1—Cl3	112.83 (2)	C16—C15—H15	119.8
Cl1—Fe1—P2^i^	88.188 (16)	C15—C16—C11	120.62 (17)
Cl2—Fe1—P2^i^	86.935 (16)	C15—C16—H16	119.7
Cl3—Fe1—P2^i^	92.918 (16)	C11—C16—H16	119.7
Cl1—Fe1—P1	87.544 (16)	C22—C21—C26	118.75 (17)
Cl2—Fe1—P1	96.663 (17)	C22—C21—P1	122.51 (15)
Cl3—Fe1—P1	88.327 (16)	C26—C21—P1	118.61 (13)
P2^i^—Fe1—P1	175.405 (17)	C21—C22—C23	120.5 (2)
N2—C1—N1	108.54 (15)	C21—C22—H22	119.8
N2—C1—H1	125.7	C23—C22—H22	119.8
N1—C1—H1	125.7	C24—C23—C22	120.1 (2)
C1—N1—C2	108.56 (14)	C24—C23—H23	119.9
C1—N1—C4	125.54 (15)	C22—C23—H23	119.9
C2—N1—C4	125.71 (14)	C23—C24—C25	119.97 (19)
C3—C2—N1	107.24 (15)	C23—C24—H24	120.0
C3—C2—H2	126.4	C25—C24—H24	120.0
N1—C2—H2	126.4	C24—C25—C26	119.8 (2)
C2—C3—N2	106.81 (16)	C24—C25—H25	120.1
C2—C3—H3	126.6	C26—C25—H25	120.1
N2—C3—H3	126.6	C25—C26—C21	120.84 (19)
C1—N2—C3	108.85 (14)	C25—C26—H26	119.6
C1—N2—C6	123.97 (14)	C21—C26—H26	119.6
C3—N2—C6	127.10 (15)	C31—P2—C41	102.89 (8)
N1—C4—C5	113.80 (14)	C31—P2—C7	106.13 (8)
N1—C4—H4A	108.8	C41—P2—C7	98.06 (8)
C5—C4—H4A	108.8	C31—P2—Fe1^i^	115.55 (5)
N1—C4—H4B	108.8	C41—P2—Fe1^i^	119.17 (6)
C5—C4—H4B	108.8	C7—P2—Fe1^i^	112.91 (6)
H4A—C4—H4B	107.7	C32—C31—C36	118.58 (16)
C4—C5—P1	114.37 (11)	C32—C31—P2	124.71 (13)
C4—C5—H5A	108.7	C36—C31—P2	116.67 (13)
P1—C5—H5A	108.7	C31—C32—C33	120.26 (17)
C4—C5—H5B	108.7	C31—C32—H32	119.9
P1—C5—H5B	108.7	C33—C32—H32	119.9
H5A—C5—H5B	107.6	C34—C33—C32	120.70 (18)
N2—C6—C7	110.95 (14)	C34—C33—H33	119.7
N2—C6—H6A	109.4	C32—C33—H33	119.7
C7—C6—H6A	109.4	C33—C34—C35	119.51 (18)
N2—C6—H6B	109.4	C33—C34—H34	120.2
C7—C6—H6B	109.4	C35—C34—H34	120.2
H6A—C6—H6B	108.0	C34—C35—C36	120.19 (18)
C6—C7—P2	114.23 (11)	C34—C35—H35	119.9
C6—C7—H7A	108.7	C36—C35—H35	119.9
P2—C7—H7A	108.7	C35—C36—C31	120.73 (17)
C6—C7—H7B	108.7	C35—C36—H36	119.6
P2—C7—H7B	108.7	C31—C36—H36	119.6
H7A—C7—H7B	107.6	C46—C41—C42	119.10 (17)
C11—P1—C21	104.64 (8)	C46—C41—P2	118.91 (14)
C11—P1—C5	104.57 (8)	C42—C41—P2	121.86 (14)
C21—P1—C5	99.29 (8)	C43—C42—C41	120.41 (19)
C11—P1—Fe1	118.44 (5)	C43—C42—H42	119.8
C21—P1—Fe1	114.14 (6)	C41—C42—H42	119.8
C5—P1—Fe1	113.56 (6)	C44—C43—C42	119.9 (2)
C12—C11—C16	118.24 (16)	C44—C43—H43	120.0
C12—C11—P1	124.42 (13)	C42—C43—H43	120.0
C16—C11—P1	117.30 (13)	C43—C44—C45	120.2 (2)
C13—C12—C11	120.75 (18)	C43—C44—H44	119.9
C13—C12—H12	119.6	C45—C44—H44	119.9
C11—C12—H12	119.6	C44—C45—C46	120.4 (2)
C14—C13—C12	120.26 (19)	C44—C45—H45	119.8
C14—C13—H13	119.9	C46—C45—H45	119.8
C12—C13—H13	119.9	C41—C46—C45	119.92 (19)
C15—C14—C13	119.60 (17)	C41—C46—H46	120.0
C15—C14—H14	120.2	C45—C46—H46	120.0
C13—C14—H14	120.2		

Symmetry code: (i) −*x*+1, −*y*+1, −*z*+1.

### Hydrogen-bond geometry (Å, º)

**Table:**

*D*—H···*A*	*D*—H	H···*A*	*D*···*A*	*D*—H···*A*
C2—H2···Cl3^ii^	0.95	2.65	3.4710 (18)	145
C3—H3···Cl1^iii^	0.95	2.80	3.406 (2)	123
C4—H4*A*···Cl3^ii^	0.99	2.98	3.6985 (18)	130
C6—H6*A*···Cl1^i^	0.99	2.77	3.4402 (17)	126
C6—H6*B*···Cl1^iii^	0.99	2.84	3.6716 (19)	143
C16—H16···Cl1	0.95	2.93	3.726 (2)	143
C32—H32···Cl1^iii^	0.95	2.91	3.6057 (17)	131
C1—H1···Cl2	0.95	2.43	3.3260 (17)	157

Symmetry codes: (i) −*x*+1, −*y*+1, −*z*+1; (ii) −*x*+3/2, *y*+1/2, −*z*+3/2; (iii) *x*, *y*+1, *z*.
