# Supplementary figures and images for: Crystal structure of [{FeCl3}2(μ-PCHP)2] [PCHP = 1,3-bis­(2-di­phenyl­phosphanyleth­yl)-3H-imidazol-1-ium] with an unknown solvent
Source: Acta Crystallogr E Crystallogr Commun. 2018 Oct 31;74(Pt 11):1686–90. doi: 10.1107/S205698901801472X (PMC6218919; doi:10.1107/S205698901801472X)

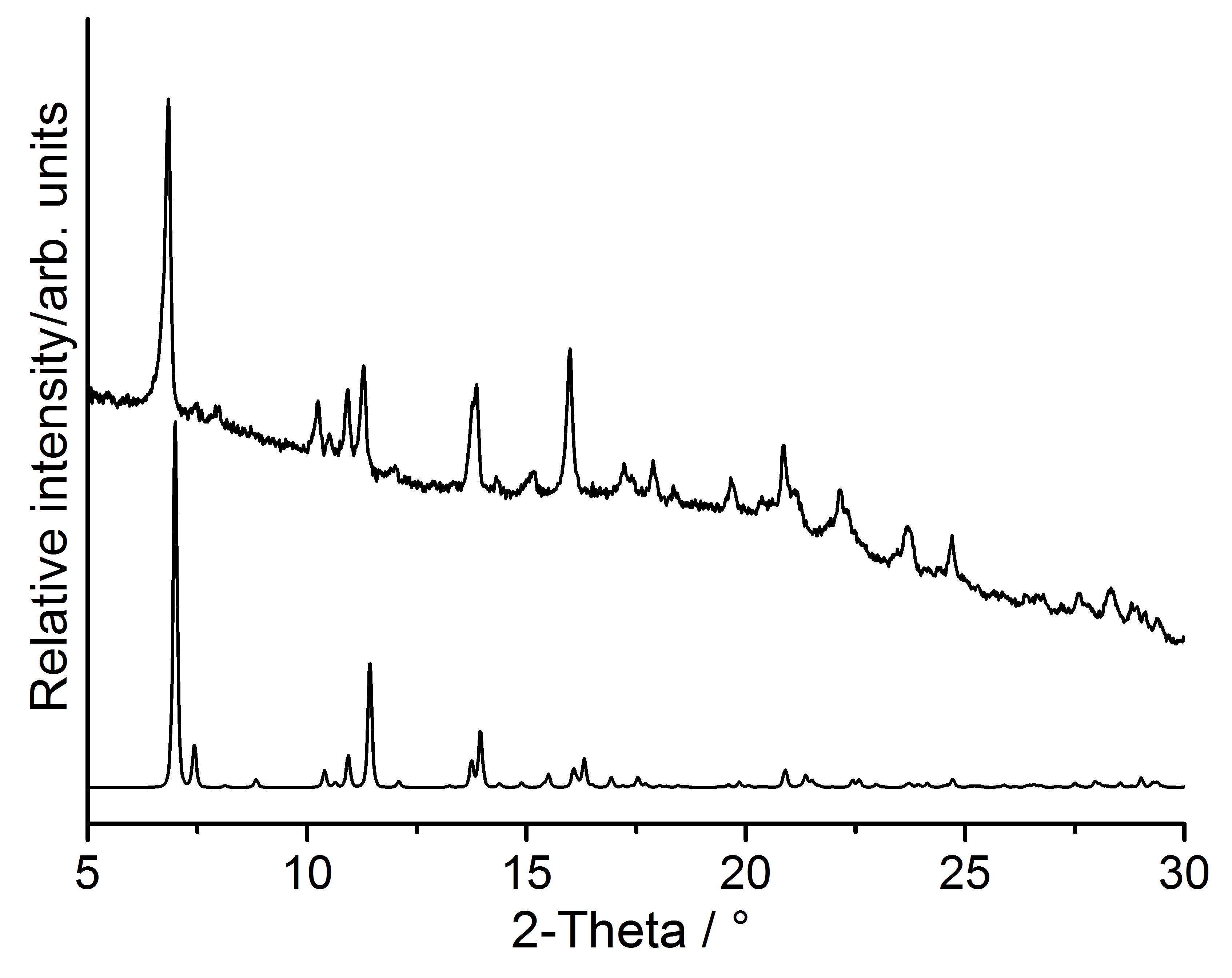

Supplement: Supplementary file 3 [file e-74-01686-sup4.jpg]
